# Ten years of negotiating rights around maternal health in Uttar Pradesh, India

**DOI:** 10.1186/1472-698X-11-S3-S4

**Published:** 2011-12-16

**Authors:** Jashodhara Dasgupta

**Affiliations:** 1 SAHAYOG, A 240, Indira Nagar Lucknow 226016, India

## Abstract

**Background:**

Preventable maternal mortality and morbidity have been globally recognized as human rights issues. Maternal mortality in India is among the highest in the world, and reflects inequity in access to healthcare: women from certain states as well as poorer women and less literate women appear to be significantly disadvantaged. The government of India has been attempting to improve maternal outcomes through a cash transfer within the National Rural Health Mission to encourage women to come to hospitals for childbirth.

**Methods:**

This paper reviews documents of the last ten years describing the experiences of a Non-Governmental Organisation, SAHAYOG, in working with a civil society platform, the Healthwatch Forum, to develop ‘rights based’ strategies around maternal health. The paper builds an analysis using recent frameworks on accountability and gendered rights claiming to examine these experiences and draw out lessons regarding rights claiming strategies for poor women.

**Results:**

The examination of documents over the last ten years indicates defined phases of development in the evolution of SAHAYOG’s understanding and of the shifts in strategy among SAHAYOG and its close allies, and responses by the state. The first three stages depict the deepening of SAHAYOG’s understanding of the manner in which poor and marginalized women negotiate their access to health care; the fourth stage explores a health system intervention and the challenges of working from within civil society in alliance with poor and marginalized women.

**Conclusion:**

The findings from SAHAYOG’s experiences with poor Dalit women in Uttar Pradesh reveal the elements of social exclusion within the health system that prevent poor and marginalized women from accessing effective lifesaving care. Creating a voice for the most marginalised and carving space for its articulation impacts upon the institutions and actors that have a duty to meet the claims being made. However, given the accountability deficit, the analysis indicates the importance of going beyond the normative to developing actor-oriented perspectives within rights based approaches, to take into account the complexity of the negotiating process that goes into claiming any kind of entitlements.

## Background

Maternal health is one of the eight Millennium Development Goals (MDGs). Its target is the reduction of maternal mortality by three-quarters between 1990 and the year 2015 [1]. Beyond global consensus that the current levels of maternal mortality are unacceptable, recent resolutions of the United Nations Human Rights Council also recognize that preventable maternal mortality is an issue of women’s human rights [2,3]. The human right to survive pregnancy and childbirth is predicated on the fact that the necessary information and technologies are available to prevent almost all maternal deaths, yet maternal deaths continue to occur in their hundreds of thousands. Preventable maternal mortality and morbidity reflect discrimination in priority-setting within health systems that leads to inequitable access to healthcare for women. Moreover, unequal gender relations prevent women from getting enough nutrition, making decisions about their fertility, and accessing contraception information and health services. Poor maternal health outcomes are influenced not just by the quality of services available, but are also a function of women’s poverty, education, location and many other socio-economic factors.

Maternal mortality in India is a significant global issue, since the country has a disproportionate share of global maternal deaths [4]. Data from recent national surveys in India indicate that the quality of services available for maternal health in rural health centres is not adequate for either routine or emergency maternal care [5]. The data also show that the poorest women, rural women and those with least literacy have the most inequitable access: they obtain far less ante-natal care and have a far lower percentage of childbirths in hospital compared to women with some education and in a higher wealth quintile. Women from certain states in northern and central India also appear to be significantly disadvantaged in this regard [5,6].

Uttar Pradesh (UP) is one such state where the maternal mortality ratio (MMR) has remained disproportionately high, at 359 per 100,000 live births, which is 1.69 times the MMR of 212 for India as a whole [7]. Since Health is a “state subject” in India, the implementation of health programmes depends largely on the political will of the state government. However, although three successive political formations have ruled UP since 2000, they paid more attention to consolidating vote-banks along caste and religious lines, and none of them took up the state’s poor health indicators as a political priority. Compounding the challenges is the vast size of the state (70 districts) and a population of close to 200 million, largely poor and rural, with 59% female literacy [8].

In congruence with the global push for the MDGs, the government of India has recently emphasized maternal mortality reduction through the Janani Suraksha Yojana (Mothers’ Protection Scheme or JSY) as part of its National Rural Health Mission, launched in 2005 [9]. The JSY includes a conditional cash transfer to encourage women to come to hospitals for childbirth, and supports a community-based link worker ‘ASHA’ or Accredited Social Health Activist [9] [p60] to ensure women register their pregnancy with the local health centres, as well as to accompany the women during labour and conduct post-partum home visits [10]. However, recent findings from civil society organizations indicate that in states with high MMR, pregnant rural women do not receive quality maternal health services at state facilities, especially if they are from lower income or lower caste groups [11,12]. As the data above has indicated, emergency obstetric care is unavailable even in the designated rural health facilities [5].

SAHAYOG is a not-for profit organization that has been working since 2000 on maternal mortality in Uttar Pradesh in partnership with a civil society alliance called Healthwatch Forum Uttar Pradesh (HF) [13]. SAHAYOG and its allies have termed maternal deaths and related ill-health a violation of ‘women’s right to maternal health’. Examining the complexity of the negotiating process that goes into claiming any kind of entitlements, this article reviews SAHAYOG’S experiences over the last ten years of working with Dalit women (formerly “untouchables” and now members of the Scheduled Castes) for improved access to maternal health services in Uttar Pradesh. It analyses the challenges of developing rights-based strategies that can enable poor women to claim accountability from the state.

## Conceptual framework

As part of a civil society alliance trying to apply rights-based approaches, there has often been a struggle between getting normative statements politically endorsed in laws or covenants, and the experience of applying them in practice. Normative statements often have limited impact on the ability of poor communities to get themselves recognized as genuine ‘rights holders’ and to be able to exercise the rights or claim state accountability for ensuring them.

Political statements of human rights such as UN treaties and laws are important at the discursive level in terms of setting global or national standards, and they symbolize a public acknowledgement of the ‘wrongs’ that prompted the codification of the ‘rights.’ But in the actual process of realizing rights, there are imbalances and complexities in the relations of power between service providers and users [14,15] or, in human rights language, the rights claimants and the duty bearers. Reviewing understandings of rights, Yamin notes that human rights language has largely maintained an understanding of human beings as autonomous individuals, without fully appreciating how social relations constitute structures of choices within which people perceive, evaluate and act [16]. A case in point is rights conferred on poor women in contexts where they have limited access to the means to actualise their entitlements and where they are often not seen, and do not see themselves, as worthy of having rights [17].

Elite biases in policy making result in skewed distribution of basic services that reflects the lack of voice of the poor in determining the types of public services that should be available, their physical location, terms of access to services, and nature of interactions between providers and users. This lack of voice, in turn, stems from a failure in representative politics. In the context of governance, ‘voice’ is understood to describe how citizens express their interests, react to governmental decision-making and respond to problems in the provision of public goods like healthcare [14].

Provider-patient relationships are also deeply influenced by the social context in which they are embedded. Where there are high levels of poverty and inequality, the social and economic status differences between providers and service users affect the capacity of poor users to obtain good quality and respectful services, and the unequal power relationship between experts and clients may be exploited by the former in their own interest [18]. The poor are likely to have to travel great distances for treatment, may be obliged to pay bribes to be seen by a health professional, may not receive appropriate treatment or may even be humiliated, and face contemptuous treatment by service providers [14].

It has been argued that the accountability and responsiveness problems among service providers are a product of difficult work conditions and an inadequate or non-existent professional service ethos. Workers who are demoralized, under-paid, and poorly resourced will seek to control the variety of demands which clients place upon them by limiting the information and services provided to socially marginal service users [14]. In fact, the poorer class of service users may be under more pressure from providers to pay bribes, because of their low social status and perceived lack of capacity to organize to expose corruption or press complaints.

Abusive treatment of service-users is enabled by failures in formal and informal accountability systems. Formal systems of administrative control, performance assessment, and grievance-redress usually fail to register or punish maltreatment of patients, citing lack of formal complaints. Poor patients on the other hand, rarely register formal complaints for fear of victimization and further abuse. In fact there are often anti-poor biases built into the direct accountability mechanisms, such as judicial remedies. When the agents claiming accountability happen to be poor and/or socially marginalised groups with few social and political resources at their command they are unlikely to be a counterweight to the considerable power of public officials and institutions. When these agents happen to be poor women the power equation can become even more unbalanced [17].

In the language of governance, the term accountability has two dimensions, *answerability*, when someone is obliged to explain their actions or decisions, and *enforceability*, when sanctions or punishments can be applied in case the answers are not satisfactory. In the practical operation of accountability systems there are two kinds of accountability: *vertical* forms, in which citizens and their associations play direct roles in holding the powerful to account, and *horizontal* forms, in which the holding to account is delegated to other powerful actors [19]. Normatively, public sector actors have a duty to be *responsive* to the members of the public with whom they interact, but are obliged to *account* for their actions only to their seniors, who are accountable upwards to the legislature and the executive, to financial auditors, and to higher court judges [14].

Vertical accountability is where the state is being held to account by non-state agents. Beyond elections, there are other processes through which citizens organize themselves, demanding explanations and threatening less formal sanctions like negative publicity. Horizontal accountability, on the other hand, consists of formal relationships within the state itself, when one state actor has the formal authority to demand explanations or impose penalties on another state actor. But there are always failures in reporting, audits, and disciplinary procedures within service bureaucracies; the checks on impropriety which should occur via the upward flow of reporting and accounting are easily undermined by collusion between superiors and subordinates.

Citizen participation has been defined as a political process that seeks to challenge exclusion from processes and decisions that affect people’s lives and health by placing a limit on power of elites to impose their own will [14]. When previously excluded or overlooked social groups demand more direct accountability, it challenges the elitist biases which may have produced their exclusion in the first place. Yamin [16] calls for distinct approaches to participation, which centrally include fostering “critical consciousness” as a precondition to effective participation. With appropriate tools and informed choices, citizens can construct claimed or demanded spaces for claiming citizenship entitlements. In this conceptualisation, participation is not merely a means by which given citizenship roles are reproduced and state obligations fulfilled, but rather, it offers the prospect that citizenship “can be claimed from below” by women and other marginalized groups “through their own efforts in organized struggles, rather than waiting for it to be conferred from above” ([16]. p16).

Goetz [20] however cautions against putting the entire onus of vigilance on those who have the least time for, and the most to lose from, challenging the local power structure on which they most likely depend, and raises ethical questions about asking those who have the fewest defences, to take the greatest risks. In a context where public officials do not see themselves as subject to accountability, and do not recognise groups of poor women as agents making moral and social claims, merely eliciting a broader expression of the voices of women will not produce changed policies or change the behaviour of bureaucrats, the police, or politicians without concurrent changes in the norms and procedures of accountability institutions.

Making women’s voices heard, respected and responded to, without the formal means to do so, thus becomes the main challenge; however, the role civil society can play is dependent to a great extent on the nature of the political system and culture, and state-society relations. As a response to these challenges, civil society organisations in India in recent years have attempted to become involved in the state’s *horizontal* accountability institutions or worked to create new institutions, or attempted to substitute for failed accountability institutions by holding their own enquiries and hearings [21]. New *‘hybrid’* forms of accountability are also emerging in a number of recent examples that suggest insights as to how citizens might prompt more satisfactory performance from authorities, as they demand *answerability* even if they do not have *enforceability*. Mukhopadhyay and Meer [17] suggest that they can do this through building strategic alliances and by drawing on the legitimacy they build up over many years as development actors in civil society.

## Methods

Using the theoretical frameworks discussed above, this paper draws upon the author’s personal engagement with SAHAYOG’s rights-based work with poor rural communities in Uttar Pradesh, as part of the post-International Conference on Population and Development (ICPD) efforts to promote reproductive rights. From 2000, SAHAYOG’s work on maternal health adopted a rights based framework, but faced a series of challenges while trying to make a difference in state apathy to high rates of maternal death in Uttar Pradesh. These were discussed in strategy planning meetings of the HF and other campaign allies. In mid-2006, the author spent a sabbatical break as Visiting Fellow at the Institute of Development Studies in the UK and engaged in a process of reflection on the last five years’ efforts and a study of the meaning and politics of Discourse Theory. Drawing from this process, SAHAYOG continued to work on maternal health using a less antagonistic approach to the state, while also engaging in regular reflection meetings every three months with all the partners and allies. Another five years ahead this is an opportune moment to reflect on an entire decade of work on maternal health rights, the adaptations through modified strategies, and the learning that emerged.

This paper is based on organizational records, including unpublished internal and external evaluation reports, in-house publications and web-based documents describing the experiences of SAHAYOG’s work of the last ten years in collaboration with allies of the Healthwatch Forum, Uttar Pradesh (HF). A significant part of SAHAYOG’s learning process has been through unpublished organizational case-files that document the struggles with actual cases of extremely poor women who faced life-threatening situations and health service denial in 2004 (Nankai, Lucknow, UP), 2007 (Manju, Lucknow, UP) and 2008 (Salenta/Snehlata, Muzaffarnagar, UP). Other key sources of information are unpublished reports of community based participatory approaches for mobilizing around the issue of women’s right to maternal health, legislative advocacy, public interest litigation, media advocacy and campaigns, and several rounds of ‘policy dialogues’.

Reflecting on the experiences of the last ten years, this paper interrogates the process of civil society action around maternal mortality in Uttar Pradesh to ask why the issue of maternal deaths never becomes a ‘political’ issue, why the agent of accountability is never clear and despite some gains at the localized sites, overall why the health system and bureaucracy remain inert; and what needs to be done differently.

## Results

Since 2000, SAHAYOG has been working on women’s right to maternal health in partnership with civil society allies. The examination of documentation over the last ten years indicates four phases in the evolution of SAHAYOG’s current understanding of the situation. These phases chart the shifts in strategy among SAHAYOG and its close allies, and responses of the state to these strategies.

### Phase one - unearthing cases and articulating maternal mortality as a rights violation

In December 2000, SAHAYOG encountered the first ‘maternal death case’ as part of a Healthwatch Forum fact-finding team visiting a small town in Uttar Pradesh. The story was narrated by the devastated husband, who explained how his wife had gone to a district hospital in July 1999 for her third childbirth and met the woman doctor on duty who gave her an injection and left her alone. As his wife developed rashes and went into distress, he made increasingly desperate efforts to get someone to attend to her, but in vain. The obstetric complication was neither detected nor managed on time, and despite having gone well in time to the best-equipped hospital in her small town, the woman died before she gave birth. The hospital took no responsibility for what had happened.

The narration of the case raised several questions: families of pregnant women have always been blamed for delay in seeking skilled care during labour, yet who was at fault when a maternal death occurred even though the woman had reached a hospital well in time? What was the injection given by the doctor, why were its effects not monitored and why was the woman not referred immediately? Could a woman doctor in a district level hospital really neglect a labouring woman to the point when her complication became fatal? If such a thing actually did happen, who was accountable? These and other questions compelled SAHAYOG and its allies to re-examine the common assumptions around the “Three Delays model” that women’s lives could be saved if women reached institutions in time during labour, and if institutions had skilled medical personnel [22]. One assumption was that skilled medical personnel would act in the best interests of the woman in labour.

The case turned out to be not an isolated one. Over the next few months, HF partners and allies unearthed a series of similar cases across several other districts of Uttar Pradesh, where the health providers appeared to be wilfully neglecting the women who attempted to reach institutions for childbirth. In some cases the providers had misdiagnosed the situation, or demanded sums of money that poor families had been unable to pay. It also emerged that the providers were hampered by lack of sufficient training, lack of essential back-up services (such as surgeons and anaesthetists) or supplies like oxygen or blood. Within a post-ICPD framework of reproductive health and rights, HF brought together many of the victims or their families to give testimonies of reproductive rights violations during a Public Hearing organized by HF at the state capital Lucknow in April 2001 [unpublished data from the event report, Healthwatch Uttar Pradesh – Bihar: Priorities of the People: People, Population Policy and Women’s Health in Uttar Pradesh; Lucknow Uttar Pradesh; March 2002]. Despite media coverage, there was no perceptible response from the government of Uttar Pradesh. In 2003, SAHAYOG began working with a group of women’s Non-Governmental Organisations (NGOs) in UP to systematically document these cases using the framework of “state accountability for women’s reproductive health and ensured maternal survival” as set out within the Convention on the Elimination of all Forms of Discrimination Against Women (CEDAW) treaty [unpublished observations by Misra S and Parbha P: An Evaluation: Women’s Voices - Monitoring Women’s Rights under CEDAW in India; SAHAYOG Lucknow; October 2004]. By early 2004, SAHAYOG had collated twelve cases, several of which involved Dalit women, and half of whom had lost their lives due to preventable causes, including being denied maternal care in institutions, or being compelled to visit multiple providers in attempts to seek care, or dying in hospitals despite having sought skilled care [unpublished data in the report SAHAYOG et al: Women’s Voices: Monitoring State Implementation of the CEDAW, an alternative report of Uttar Pradesh state on Women’s Health and Violence Against Women; SAHAYOG, Lucknow; 2004].

These cases were used in media briefs during a state-wide campaign called Complete Citizens Total Rights-I campaign [23] by women’s organizations in late 2003 that posited maternal deaths as a violation of Constitutional guarantees to life and health, and tantamount to lack of equal citizenship rights for women**.** On 10 December (Human Rights Day) activists demonstrated, dressed as corpses, to draw attention to rights violations leading to preventable maternal deaths. On 8 March, 2004 (International Women’s Day), the campaign demands were presented to the Uttar Pradesh Chief Minister, and fortuitously followed by a government announcement to start maternal death reviews in the state. However, no effective action followed on the part of the government beyond a small pilot effort that elicited total denial of any maternal deaths from the district officials concerned.

### Phase two - beginning to engage with state actors

From 2004, SAHAYOG attempted to modify the original tone with the state on the grounds that it was too accusatory and began facilitating a process of regular dialogue of the NGOs and rural women leaders with state actors. A state-civil society dialogue on maternal health was organized by HF on the International Day of Action on Women’s Health on 28^th^ May 2004, a practice which has continued regularly over seven years to bring rural women to interact with policy actors at the state capital**.** Despite this, cases of negligence and preventable maternal deaths continued to surface. In July 2004, there was a media report about a poor Scheduled Caste or *Dalit* (the ‘untouchable caste’) woman, Nankai, who had come to a hospital for her first childbirth. She was abused by the nurses and attendants as she couldn’t pay enough money in informal fees, and actually thrown out of the hospital during labour. She gave birth in the open outside the gate, and the baby died within a short while. The activists of HF worked with lawyers, the media and the local Member of Legislative Assembly to help Nankai to access medical care and justice. She had developed a vaginal tear that got severely infected and required a month of hospitalization. HF’s case-file notes (2004) indicate that senior government doctors were colluding to conceal medical evidence on the case, and despite petitions to all concerned officials including the Health Secretary, the health department refused to take any systemic action beyond punishing a nurse in the hospital.

Nankai finally found support from the Uttar Pradesh Commission for Prevention of Atrocities on Scheduled Castes and Tribes, which recommended that she be compensated for the death of her baby, and instituted criminal proceedings against the doctor on duty. But the criminal case in court was too prolonged for the impoverished family to pursue: after a year they succumbed to the doctor’s offer of a small financial settlement. The year-long media campaign around this incident starkly highlighted the reality of poor and Scheduled Caste patients who are harassed for informal fees in hospitals, and which the print media covered constantly for the next few years [unpublished observations of Solanki, P: Report of the Evaluation of Media Advocacy on Maternal Health by SAHAYOG (2006-2009), SAHAYOG, Lucknow; September 2009]. This case, and many others after it, exemplified the elements of discrimination and social exclusion within the health system that prevent poor and marginalized women from accessing effective lifesaving care.

The National Rural Health Mission (NRHM) was announced in Uttar Pradesh in September 2005, and institutional delivery within the Mothers’ Protection Scheme (JSY) was promoted as the answer to high rates of maternal death [9]. Public health expertise had located the roots of the maternal mortality problem as a ‘demand-side issue’, thus the state had instituted community animators such as the ASHA to motivate pregnant women to register themselves, and come to the government facilities during labour. But the struggles around Nankai’s case had already indicated to SAHAYOG and HF that despite the JSY, the health institutions in Uttar Pradesh would not guarantee quality care to poor women, and that there was no mechanism to ensure some degree of accountability for this. In the meantime, the government orders for maternal death reviews died an unnoticed death since district medical officers sent in reports of zero maternal mortality each month. It appears also that the health department never got round to constituting the State Committee that would study these reports.

SAHAYOG and the allies of HF began examining women’s experiences within the new NRHM framework. Initially working with six partner NGOs across the state in 2005-2007, SAHAYOG collated another 23 post-NRHM cases of maternal deaths and near-miss situations [12]. Despite the women attending hospitals during labour, they had faced poor quality services, petty corruption and in some cases outright denial of care. The cases once again raised the basic questions of institutional capacity and willingness to respond to poor women’s increased demand for services. The findings from these cases were provided as civil society feedback on the implementation of the NRHM [12].

In 2006, SAHAYOG, in partnership with HF and many other civil society groups began another state-wide campaign (Complete Citizens Total Rights-II campaign) to address policy makers, constructing preventable maternal mortality as lack of equal citizenship for women. Once again women demonstrated, dressed as corpses and tried to focus media attention on the problem. The campaign mobilized thousands of rural women across half the state; and some of them presented their experiences to the Health Minister and spoke before legislators [24]. It is worth noting that the political class did not pay much attention to the women’s issues and recommendations although they presented themselves as the constituency that provides the votes that elect leaders into power. The Director General of the Health and Family Welfare department, however, did take their feedback more seriously and issued government orders accordingly.

SAHAYOG and its allies were faced with the question of who is the appropriate ‘duty bearer’ responsible for the rights holders’ (women’s) access to quality health services that could save their lives? There appeared to be a bewildering array of those who were normatively responsible: dealing with policies, programmes and service provision (but none apparently dealing with grievance redress). Initially the women had addressed policy actors, such as the Health Minister, and Director of the Health and Family Welfare department during their campaign. Yet this did not lead to perceptible improvements on the ground, so the modified strategy was to directly address the programme managers within the districts, as well as engage with the local frontline workers.

Although the state-wide campaign enabled women to clearly articulate their rights to health, the one-off efforts did not lead to the expected state response from the political actors. It became evident that more sustained collective efforts were needed to continue campaigning, especially to bring forward the voice and experiences of Dalit women and similar marginalized groups. The campaign mobilization in 2006 led to a change in strategy in favour of creating an “organization of the rights holders”, thereby constructing a civil society that is inclusive and capable of representing the interests of women from marginalised groups, that enhances poor women’s participation and agency in the process of rights-claiming, and seeks responsiveness from governance institutions.

### Phase three – building voice and participation of the user community

In May 2006, the decision was taken by women leaders and civil society groups to form a grassroots women’s organization in Uttar Pradesh, called the Women’s Health Rights Forum (MSAM for Mahila Swasthya Adhikar Manch). The MSAM organization was in a sense the political response of the beneficiary group (users of state health services) to the provisions for entitlements within the National Rural Health Mission. Additionally, the NRHM provided a legitimized space for the active engagement of the MSAM ‘rights holders’ as it had the mandate for decentralized planning and budgeting, citizen representation on oversight committees, as well as citizen monitoring.

In partnership with the MSAM and the facilitating NGOs within ten districts of Uttar Pradesh, SAHAYOG proceeded to build local women’s capacities in relation to maternal health issues. The methodology was an adaptation of ‘conscientization’ methodologies developed by Paolo Freire [25] to examine the issue of preventable maternal mortality as a violation of women’s basic rights. This has resulted in a shift in consciousness wherein the women leaders of MSAM have understood that maternal deaths are not just a matter of fate or misfortune but a matter of social injustice and that the state has a role to play in the remedy.

Armed with information about their entitlements and state provisions, the MSAM women, in an exercise of ‘active citizenship’ through monitoring and advocacy, took up various aspects of the NRHM each year for interrogation. At the start they revealed local corruption in the appointment of the ASHA workers (2006), then they audited the payment of the conditional cash transfer under the JSY (2007-8); they examined how ‘untied’ health budgets are spent locally and how much poor families are spending (2009), and recently they audited the compliance of health sub-centres with the Indian Public Health Standards (2010). Every NRHM monitoring exercise was followed by a formal presentation to the district health officials by the NGOs and MSAM women, as well as presentations at the state capital, Lucknow, usually in the presence of state officials and the media [26].

In addition, the NGOs and the MSAM continually identified cases of maternal deaths and ‘near-miss’ situations that were collated by HF. The individual ‘claims process’ itself required resources that poor women lack; as such, SAHAYOG and allies decided to move away from conventional human rights work that often engages in litigation for individual cases, and attempted to foster a ‘collective claims process’. In 2008, MSAM women presented a list of recent cases to the Health Minister; and maternal death cases were presented twice at Public Dialogues in 2009 [27,28], one panel of which included a representative of the State Human Rights Commission (SHRC). The SHRC responded by setting up a committee to investigate maternal mortality as a human rights issue, including HF as a key civil society member. The Health Minister also appears to have instituted some enquiries into the cases.

The grassroots feedback provided by the MSAM during the dialogues at Lucknow led to government orders being issued from the health department, while the regular dialogue at district level has also led to some recognition of the MSAM and the supporting NGO, towards more opportunities for involvement within the NRHM implementation, in their district. There are certainly local improvements in health service provision for villages where the MSAM is active. In some districts the officials responded to the women’s feedback by providing the MSAM leaders with their personal cell-phone numbers as a sort of hotline in case they need to get in touch for an emergency.

Since its inception in 2006-2007, MSAM membership has gone up by 2010 to 11,000 members across ten districts, with three tiers of elected leadership in the form of teams working on health and social determinants. Women’s rights articulation and claiming has expanded into livelihood and food security entitlements. In 2010, the MSAM participated in local council (Panchayat) elections and won in two-thirds of the seats they contested [29]. The largely rural and non-literate MSAM women have of course had the support of local NGO partners of SAHAYOG, as well as a civil society District Forum, including lawyers, journalists, health providers and activists.

The unrelenting media coverage of corruption in hospitals, maternal and infant deaths and the dysfunctional aspects of the health system over the last six years, occasionally spurred the health department to take some action, though usually against the lowest cadre of staff. According to media reports, the Chief Minister has sporadically urged senior officials to be more vigilant and carry out field visits. Yet there appeared to be little discussion on systemic or structural changes needed. On its own, the UP government health department has been slow in engaging with NGOs or development partners on maternal health, although this has recently improved.

When civil society organizations, speaking on behalf of the poor initially mediated the rights-claiming to address powerful policy actors such as the Chief Minister, it did not stimulate any accountability mechanism within the state to address the issue. Neither is there any noticeable systemic improvement after the organized group of rights-holders themselves addressed three consecutive health Ministers (2006, 2008 and 2010). The cases of preventable death, denial of care and demands for money have continued with almost total impunity for several years. They have continued even following the announcements about the JSY; both within health facilities that had capacity to provide the services, and those without.

### Phase four – using research

Discussions about the NRHM in 2007 with civil society groups and researchers in other Indian states revealed considerable similarities in poor women’s experiences of seeking care during pregnancy, and a similar concern with the implementation of the JSY strategy. Towards building evidence around these questions, SAHAYOG anchored a collaborative voluntary block-level study across six states of India (including Uttar Pradesh) in 2008 on poor women’s experiences with attempting to seek institutional care during childbirth. The draft findings of the study were formally presented in 2009 to various policy players at the national level [30], and in 2010 at a global maternal health conference [31], using quality of care frameworks and cost analyses to build a case for re-examining the implementation of the JSY. The qualitative findings as case studies were also shared through a simple Hindi booklet for rural women in Uttar Pradesh to read out and discuss at village meetings [32], towards building a set of recommendations which the MSAM presented to the UP Health Minister in 2010.

Meanwhile in 2008, SAHAYOG also began experimenting with a new approach to address the various stakeholders involved with maternal health in UP. Recognizing that earlier interventions had addressed the State as monolithic, a more nuanced approach was taken up through an action research project, which acknowledged that the State was composed of various stakeholders with diverse perspectives and capacities; such as political actors who relied on popular support from the ‘user community’ as well as programme managers and implementers, facility managers, frontline providers and community volunteers. An action research intervention was designed for coalition-building among stakeholders, towards building a consensus that the high rate of preventable maternal mortality in Uttar Pradesh was unacceptable.

The action research project began in 2008 in five districts with a ‘stakeholder assessment’ that mapped the positions of various stakeholders on the issue of maternal mortality; the findings from which enabled SAHAYOG to develop some understanding of the differences in perspective, and the difficulties in bringing about meaningful change in maternal health service provision. The respondents who were either managing the district health programmes or health facilities, or providing services, felt poor maternal health was due to shortcomings within the community such as poor diet, ignorance and delayed care-seeking. The major barriers they identified included massive staff shortages, and political interference [33]. These perspectives were very different from those of poor rural women who attempted to access services, and were often faced with denial or poor quality care, or harassment for money.

The preliminary findings of this perspective-mapping exercise were then shared through district dialogues in two districts to facilitate discussion among the stakeholders. Within a continuum of interactions and trust-building, these sharing meetings enabled the health managers, providers, users and NGOs to discuss the problems related to provision of maternal health services without expressing mutual antagonism. This negotiating strategy was enhanced through positive communications on maternal health using a quarterly Hindi newsletter. The facilitation of this process led to building a relationship of trust with the district health officials, due to which they permitted SAHAYOG to organize a study tour in 2010 for providers from one district to the other to see how a model PHC could improve maternal health services.

But this relationship could not be extended beyond the districts: when the research project was presented to the UP Health Department, the Director conveyed his assent and assured full cooperation. When actually requested to give formal permission, the Director delayed it for over three months and finally declared that the application had been sent up to the Secretary for approval. The departmental inability to grant permission to an NGO for a small action research study in five districts was revealing about its level of autonomy. Likewise when SAHAYOG attempted to seek permission to take a few district health officials on a study tour to Tamil Nadu to learn about maternal death audits, there was initially lack of clarity as to who was the final authority to grant permission. Repeated applications to various officials in the health department, the NRHM management unit and the Health Secretary over a period of two months finally culminated in a refusal of permission for the tour to go ahead.

In a continued effort to engage with the state in a technical capacity, SAHAYOG then began strategically working with UNICEF to promote the formation of a platform of development partners and NGOs working within Uttar Pradesh on maternal, newborn, child health and nutrition (MNCHN), in an attempt to re-activate the Technical Support group with the government. The MNCHN Partners’ Forum includes some international NGOs, national organizations and local NGOs, who have been working together for almost two years. They have made recommendations for the NRHM State Plans for UP (2010-2011 and now 2011-2012) and opened a renewed process of dialogue with the health department for a collaborative monitoring of the government maternal health programme.

## Discussion

The experience of SAHAYOG and its allies in Uttar Pradesh shows some of the challenges of civil society in mobilising the voice of affected groups towards enhancing state accountability, without any formal authority or power to do so. Despite Uttar Pradesh having the highest numbers of maternal deaths in India, formal systems of administrative oversight have not so far monitored the extent of adverse outcomes for pregnant women who enter the health system. When women’s adverse experiences and outcomes are actually reported, there is no grievance redress available. Responsiveness is displayed only by local officials and guardianship institutions so far, and even that has limited impact. In this context of accountability failure within state institutions, the question that arises is how affected populations and civil society can work to claim accountability for basic services like maternal healthcare.

The experiences of SAHAYOG and its allies question assumptions about state-civil society relations implicit in ‘rights-based approaches’ advocated by international development organizations. At the outset, SAHAYOG and its allies took up the cause of maternal health using the rights-based frameworks that are in the international development discourse. However, the normative language around state accountability is situated in an imagined context of state-civil society relations, where the state is imagined as a ‘singular and sovereign adjudicator and enforcer of rights’ [34] and where civil society can and does hold the state to account. But an actor-oriented perspective calls this into question: not all rights holders can become ‘claimants’ and in fact may not have the confidence and resources to get into the claiming process at all, being already disadvantaged by gender, race, caste, location and other power differentials [21,35]. As Mukhopadhyay and Meer [17] point out, public officials do not see themselves as subject to accountability and do not recognise groups of poor women as agents making moral and social claims of the powerless on the powerful.

The other question that arises is why the government remains unable to regulate and improve health services in Uttar Pradesh, although localized improvements in service provision have been observed as a result of pressure from the MSAM. The failure of public services to meet the expectations of the poor has been amply discussed by Goetz and Jenkins [14], who delineate the roles of “bias and corruption” in denying essential needs to poorer populations with less ‘voice’, especially where the middle class has itself opted out of state-provided services, and does not call for state accountability.

Although in recent years the NRHM attempted to inject some energy into maternal mortality prevention through the JSY and the ASHA programme, the concurrent strengthening of the dysfunctional health system in UP did not have as much priority. This was possibly influenced by the national policy diagnosis that high maternal mortality was basically a ‘demand-side problem’, which could be solved by ‘demand-side financing’ for getting pregnant women into hospitals. The lack of skilled personnel to staff the rural health centres has been a long-standing problem that cannot be solved by getting short term contractual workers. The bottlenecks with staff postings and transfers, like logistics and procurement, are clogged with massive corruption that has the connivance of the political class. Substantial improvements in the quality of services will require enormous political will as well as some amounts of lateral thinking about how to solve the chronic problems.

The recent civil society reports on maternal health services for women who belong to socially marginal groups [11,12,30,31] and the testimonies of poor women in recent public hearings in Uttar Pradesh [27,28] are also revealing in terms of how social exclusion operates within health systems. In addition to the health system issues discussed above, the duty bearers appear to hold a world view that precludes seeing Dalit and other disadvantaged women as human beings of equivalent worth: you can in fact die even after reaching a well-resourced institution if you are likely to be turned away or harassed for money and denied care.

This indicates that we need to contextualise, within the current matrix of social power relations, the normative rights that are apparently enshrined in Patients’ Rights Charters or Concrete Service Guarantees or in spaces designed for citizen participation. The informed interrogation of the duty bearers by a hitherto voiceless community is an important shift from passive to active voice. But voice without authority, may only raise demands for accountability: this does not automatically translate into answerability.

## Conclusions

Over ten years, SAHAYOG has built a wide array of alliances in civil society and change strategies for mobilisation to address the state and to make public servants accountable. But the issue of high maternal deaths in Uttar Pradesh never becomes a ‘political’ issue, the agent of accountability is never clear and despite some gains at the localised sites, overall the health system and bureaucracy remain inert. An analysis of the civil society strategies and state responses based on the theoretical framework of this paper indicates some ways forward for operationalising the rights of poor women.

The state is not a monolith: the diversity of state and non-state actors needs to be mapped out to find entry points through exploiting this diversity and identifying more responsive state institutions. Taking up cases of maternal health rights violations with the various commissions for the protection of human rights and the judiciary that are supposed to act as guardianship institutions can further create “hybrid spaces” where civil society engages with a horizontal accountability institution. Possibilities remain open for local and provincial engagements through providing technical support to the Uttar Pradesh state health department. Civil society organisations can insert themselves within state processes, and through working on the inside bring women’s voices to state processes and actors, even without the formal authority to do so.

SAHAYOG and its allies needed to reconsider earlier antagonistic approaches in favour of a more nuanced consideration of the differing perspectives and constraints under which duty bearers work, including the ambiguity about their roles and levels of authority that pervades many levels of the health department, discouraging the managers from taking initiative for problem-solving. Given the accountability deficit within UP, developing sites for *institutionalised co-production* may be the way forward, where the informed consumer can also play a part in the regulation of service providers.

The MSAM organization as ‘informed interlocutors’ of state health services has fostered the rural women’s discursive claims, such as the claim to have a voice, to be heard and to participate in decisions that affect one’s life. When poor women articulate their claim to state accountability for their sisters who lost their lives, it brings an element of social justice into reviewing the quality of the outcomes of public decisions and actions: accountability can no longer be seen as a merely managerial function of assessing whether processes are diligently followed.

The recent entry of the MSAM into local elected councils (Panchayats) has the potential to facilitate their engagement with local arenas of decision-making, although it is as yet inadequate to influence larger structural improvements or bring about improvements in public institutions’ response to poor women. Influence in local councils runs the risk of being limited to the local delivery of health programmes, while key decisions that take place at a district or central level relating to resource allocation, health care workforce, or structuring of health systems are never “up for contention”.

Creating a voice for the most marginalised and carving space for its articulation impacts upon the self-esteem of the claimants and on their appreciation of the ‘right to have rights’, as well as on the institutions and actors that have a duty to meet the claims being made. The informed interrogation of the state by a hitherto voiceless community is an important shift from passive to active voice. However, given the accountability deficit, civil society will have to continue exploring new strategies until quality health services are universally available to the poor.

## List of abbreviations

ASHA: Accredited Social Health Activist; CEDAW: Convention on the Elimination of all Forms of Discrimination Against Women; HF: Healthwatch Forum (Uttar Pradesh); JSY: Janani Suraksha Yojana (Mothers’ Protection Scheme); ICPD: International Conference on Population and Development, 1994; MDGs: Millennium Development Goals; MMR: Maternal Mortality Ratio; MNCHN: Maternal, Newborn, Child Health and Nutrition; MSAM: Women’s Health Rights Forum (MSAM for Mahila Swasthya Adhikar Manch); NGOs: Non-Governmental Organisations; NRHM: National Rural Health Mission; PHC: Primary Health Centre; SHRC: State Human Rights Commission; UNICEF: United Nations Children’s Fund; UP: Uttar Pradesh.

## Competing interests

None.
